# ETHICS AND ACCEPTANCE OF AI

**DOI:** 10.1093/geroni/igae098.0911

**Published:** 2024-12-31

**Authors:** Shelia Cotten, Aishah Khan, Catherine Mobley, Xia Jing, Jihad Obeid, Grant Goodrich, Hamilton Baker, Nina Hubig

**Affiliations:** Clemson University, Clemson, South Carolina, United States; Clemson University, Clemson, South Carolina, United States; Clemson University, Clemson, South Carolina, United States; Clemson University, Clemson, South Carolina, United States; Medical University of South Carolina, Charleston, South Carolina, United States; Medical University of South Carolina, Charleston, South Carolina, United States; Medical University of South Carolina, Charleston, South Carolina, United States; Clemson University, Clemson, South Carolina, United States

## Abstract

Over the past 18 months, artificial intelligence (AI) applications have proliferated and been disseminated across wider audiences than in prior time periods. AI tools are increasingly being used as part of new biomedical devices and may become more integrated into healthcare processes over time. While AI applications may have significant value in healthcare and helping support older adults and individuals across the life course through a variety of mechanisms, if individuals do not perceive these tools as ethical and are not accepting of these new tools, they are likely to fail. Cotten and colleagues report the results of a scoping review designed to assess stakeholder (e.g., patients and healthcare providers) acceptance and willingness to use AI-enabled devices in diagnosis, treatment, and rehabilitation settings. The results of this study identify perceived challenges of integrating AI into healthcare across stakeholder groups and provide suggestions to help advance AI acceptance and reduce ethical concerns.

